# 3D printing in neonatal care

**DOI:** 10.1186/1824-7288-41-S1-A1

**Published:** 2015-09-24

**Authors:** Roberto Aufieri, Simonetta Picone, Maurizio Gente, Piermichele Paolillo

**Affiliations:** 1 Division of Neonatology and Neonatal Intensive Care, Casilino General Hospital, Roma, Italy; 2 Department of Pediatrics and Infant Neuropsychiatry, Neonatal Emergency Transport Service, Sapienza University of Roma, Roma, Italy

## 

In recent years additive manufacturing, or three-dimensional (3D) printing, it is becoming increasingly widespread and used also in the medical and biomedical field [1].

3D printing is a technology that allows to print, in plastic or other material, solid objects of any shape from its digital model. The printing process takes place by overlapping layers of material corresponding to cross sections of the final product. The 3D models can be created *de novo,* with a 3D modeling software, or it is possible to replicate an existing object with the use of a 3D scanner. In the past years, the development of appropriate software packages allowed to generate 3D printable anatomical models from computerized tomography, magnetic resonance imaging and ultrasound scans [2,3].

Up to now there have been 3D printed objects of nearly any size (from nanostructures to buildings) and material. Plastics, metals, ceramics, graphene and even derivatives of human tissues. The so-called “bio-printers”, in fact, allow to print one above the other thin layers of cells immersed in a gelatinous matrix. Recent advances of 3D bioprinting enabled researchers to print biocompatible scaffolds and human tissues such as skin, bone, cartilage, vessels and are driving to the design and 3D printing of artificial organs like liver and kidney [4].

Dentistry, prosthetics, craniofacial reconstructive surgery, neurosurgery and orthopedic surgery are among the disciplines that have already shown versatility and possible applications of 3D printing in adults and children [2,5]. Only a few experiences have instead been reported in newborn and infants. 3D printed individualized bioresorbable airway splints have been used for the treatment of three infants with severe tracheobronchomalacia, ensuring resolution of pulmonary and extrapulmonary symptoms [6,7]. A 3D model of a complex congenital heart defects have been used for preoperative planning of intraoperative procedures, allowing surgeons to repair a complex defect in a single intervention [8].

As already shown for children with obstructive sleep apnea and craniofacial anomalies [9]. personalized 3D printed masks could improve CPAP effectiveness and comfort also in term and preterm neonates.

Neonatal emergency transport services and rural hospitals could also benefit from this technology, making possible to print medical devices spare parts, surgical and medical instruments wherever not readily available.

It is envisaged that 3D printing, in the next future, will give its contribute toward the individualization of neonatal care, although further multidisciplinary studies are still needed to evaluate safety, possible applications and realize its full potential.
